# Iridium-Complexed Dipyridyl-Pyridazine Organosilica as a Catalyst for Water Oxidation

**DOI:** 10.1021/acs.inorgchem.3c01386

**Published:** 2023-07-17

**Authors:** Raúl Rojas-Luna, Juan Amaro-Gahete, César Jiménez-Sanchidrián, José Rafael Ruiz, Dolores Esquivel, Francisco José Romero-Salguero

**Affiliations:** Departamento de Química Orgánica, Instituto Químico para la Energía y el Medioambiente (IQUEMA), Facultad de Ciencias, Universidad de Córdoba, Campus de Rabanales, Edificio Marie Curie, E-14071 Córdoba, Spain

## Abstract

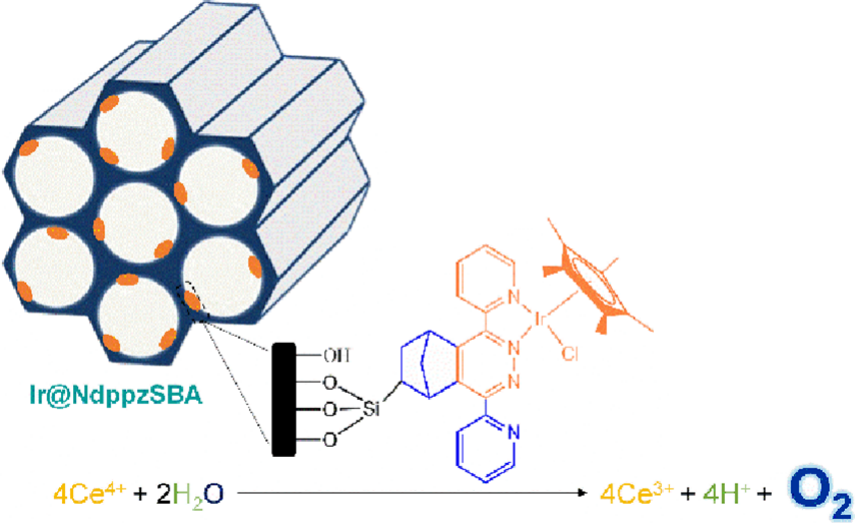

The heterogenization of metal-complex catalysts to be applied in water oxidation reactions is a currently growing field of great scientific impact for the development of energy conversion devices simulating the natural photosynthesis process. The attachment of IrCp*Cl complexes to the dipyridyl-pyridazine N-chelating sites on the surface of SBA-15 promotes the formation of metal bipyridine-like complexes, which can act as catalytic sites in the oxidation of water to dioxygen, the key half-reaction of artificial photosynthetic systems. The efficiency of the heterogeneous catalyst, Ir@NdppzSBA, in cerium(IV)-driven water oxidation was thoroughly evaluated, achieving high catalytic activity even at a long reaction time. The reusability and stability were also examined after three reaction cycles, with a slight loss of activity. A comparison with an analogous homogeneous iridium catalyst revealed the enhanced durability and performance of the heterogeneous system based on the Ir@NdppzSBA catalyst due to the stability of the SBA-15 structure as well as the isolated metal active sites. Thereby, this new versatile synthesis route for the preparation of water oxidation catalysts opens a new avenue for the construction of alternative heterogeneous catalytic systems with high surface area, ease of functionalization, and facile separation to improve the efficiency in the water oxidation reaction.

## Abstract 2

Iridium-based heterogeneous water oxidation catalysts (WOCs) for the design of efficient artificial photosynthesis systems.

## 1 Introduction

Catalytic water oxidation (WO) to molecular oxygen has gained special relevance in recent years as a key half-reaction in artificial photosynthesis systems applied to water splitting for solar fuels production.^1^ Photosystem II of oxygenic organisms (plants, algae, and cyanobacteria) performs this four-electron process (eq 1) with a Nernstian potential of 0.82 V vs NHE at neutral pH and plays a crucial role in the overall photosynthesis:
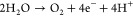
1

The generated protons are subsequently transferred to Photosystem I for the production of hydrogen, whereas the reducing equivalents are stored in the form of energy-dense carbon fuels such as carbohydrates through the carbon dioxide fixation. Therefore, overcoming the thermodynamic and kinetic barriers established for this reaction continues to be the main challenge for the scientific community. In fact, the WO reaction is the rate-limiting step in water splitting.

As nature uses a Mn_4_CaO_5_ core to catalyze this reaction, different researchers have specifically studied the structure and reaction mechanism of this multimetal complex,^2^ believing that only multinuclear metal centers were the exclusive water oxidation catalysts (WOCs) useful for this reaction.^3^ These multimetal oxidation catalysts were able to facilitate the distribution of the four oxidizing equivalents and stabilize the WOC intermediates during the catalytic reaction. Among this series of WOCs, several molecular complexes and molecular assemblies based on tetramanganese,^4^ dimanganese,^5^ tetracobalt,^6^ tetraruthenium,^7^ and diruthenium complexes^8,9^ have been particularly relevant. Likewise, heterogeneous catalysts incorporating nanostructured metal oxides (Mn, Co, Fe, Ru, and Ir) have been also reported to split water effectively.^10−14^

During the last decades, different studies have reported the catalytic oxidation of water occurring at a single metal site, including noble metals such as Ru and Ir and earth-abundant non-noble metals such as Fe, Co, and Mn.^15−20^ Single-site WOCs have attracted great scientific expectation possibly due to the following: (i) atom economy compared to multinuclear metal complexes; (ii) simplicity in the synthesis and characterization of single-nuclear metal complexes and ligand design; (iii) ligand effects in stability and catalytic activity can be easily adjusted; (iv) the geometric and electronic structures of mononuclear WOCs and their relationship with catalytic performance could be systematically investigated; (v) kinetic studies are relatively simple based on their well-defined spectroscopic and electrochemical characteristics; and (vi) the possibility of preparing heterogeneous molecular assemblies or platforms integrating catalytic functions of a single-metal site and other desired properties to build improved devices for water oxidation.^21^

Cerium(IV) ammonium nitrate (CAN) is a one-electron oxidant capable of mimicking the multiple, sequential, and single-electron transfer processes that occur in natural photosynthesis schemes. The use of CAN in the water oxidation reaction (WOR) (eq 2) is attractive because of its commercial availability, its long stability in aqueous solutions, and its absorption only in the UV range, making it suitable for monitorization of the oxidant consumption during the reaction. Moreover, CAN possesses a large redox potential of 1.61 V vs NHE, so its oxidation potential is more positive than conventional WOC for water oxidation, making the reaction thermodynamically favorable.
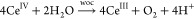
2

Therefore, CAN is a suitable oxidizing agent for iridium water oxidation catalysts.^22−24^ Mechanistically, Ir^V^-oxo species are generated as intermediates in the presence of CAN. Subsequently, the formation of the O–O bond occurs through a high-oxidation state metal oxo center according to two different pathways^17,25−28^: (i) water nucleophilic attack (WNA) to Ir = O species leading to the formation of iridium hydroperoxide, Ir-O-O-H and (ii) interaction of two Ir-O units (ROC, radical oxo coupling) generating Ir-O-O-Ir species. Thus, oxygen evolution occurs with the subsequent release of O_2_ from the intermediates. The first homogeneous mononuclear iridium water oxidation catalyst was reported in 2008 by Bernhard et al.^29^ The catalyst [Ir(ppy)_2_(OH_2_)_2_]OTf (ppy = phenylpyridine) showed remarkable activity, achieving a theoretical maximum yield of 430 μmol of O_2_ after approximately 10 h (172 mM CAN, 1.46 μmol WOC). To improve the O_2_ evolution activity, Crabtree et al. incorporated a more electrodonating ligand, pentamethylcyclopentadienyl (Cp*), to stabilize the high-valent intermediates required for water oxidation, in addition to the 2-phenylpyridine or 2-phenylpyrimidine and 2,2’-bipyridine ligands, in the coordination sphere of Ir^3+^ in a new type of iridium mononuclear [IrCp*] water oxidation complexes.^17,25^ Although this homogeneous complex [IrCp*] exhibited high catalytic activity in water oxidation, its main drawback was its poor stability under strong oxidizing and acidic reaction conditions due to the degradation of Cp* ligands to form short-chain organic acids.^30,31^ Therefore, the immobilization of [IrCp*] onto a heterogeneous support is a highly interesting research topic for a future industrial scale-up application.^32^ In 2011, Lin et al. developed the first heterogeneous porous assembly in which a IrCp* complex was attached into the pore surface and used as a water oxidation catalyst (WOC).^33,34^ Thus, the IrCp* complex was coordinated to the bipyridine chelating ligand of the UiO-67 MOF building units (Ir-bpy-MOF). The catalytic activity of the system (initial TOF= 0.12 min^–1^) was about 40 times smaller than the homogeneous one under the same conditions (ca. 3 mM CAN), which could be attributed to limitations in CAN diffusion (molecular size ∼1 nm) through the MOF channels due to its small pore size (∼1 nm) and its poor stability under high CAN concentration. In the following years, Inagaki et al. designed several scaffolds based on mesoporous organosilicas to enhance the catalytic activity of heterogeneous IrCp* complexes as WOCs. Among all the mesoporous hybrid materials synthesized, organosilica nanotubes with phenyl and 2,2’-bipyridine bridged organosilane precursors stood out because of its high stability and large pore diameter, which facilitated CAN transport through the channels (initial TOF = 3.1 min^–1^).^35^ In addition, three Ir complexes fixed on the surface bridges of a 2,2’-bipyridine periodic mesoporous organosilica (Ir_*x*_-Bpy-PMO, *x* = 0.03, 0.07, and 0.16 Ir/bpy molar ratio) also showed high catalytic activity (initial TOF = 2.8, 2.5, and 2.1 min^–1^, respectively), although a collapse of the pores with continuous reuse cycles was reported.^36^ Furthermore, they synthesized three new iridium-based mesoporous hybrid materials in which mesoporous organosilica nanotubes (NTs) and SBA-15 mesoporous silica containing bipyridine branched-chain bis-silane precursors were used to coordinate Ir active sites. The obtained Ir-Bpy-NT, Ir-Gbpy-NT, and Ir-Bpy-SBA-15 materials were evaluated as potential water oxidation catalysts showing TOF during the initial 15 min of 1.2, 0.8, and 0.6 min^–1^, respectively.^37^

Herein, we report the synthesis of a heterogeneous water oxidation catalyst based on an SBA-15 material via grafting of a dipyridyl-pyridazine triethoxysilane precursor onto the silanol groups of the silica surface and subsequent complexation of iridium by postsynthetic metalation. The performance of this heterogeneous WOC (Ir@NdppzSBA) has been tested in chemical water oxidation using CAN as an oxidizing agent, including its activity and stability after reaction. This single-site solid catalyst showed high catalytic performance at long reaction times and was efficiently reused for three cycles, with only a slight loss of activity. Furthermore, the homogeneous iridium complex was also evaluated as WOC, giving rise to a lower performance in the oxygen evolution reaction than the heterogeneous catalyst Ir@NdppzSBA, thus demonstrating the critical importance of the iridium immobilization on a mesoporous silica support with a large pore size as SBA-15 to overcome the diffusion limitations of the reactants and products during the reaction.

## 2 Results and discussion

**Scheme 1:**
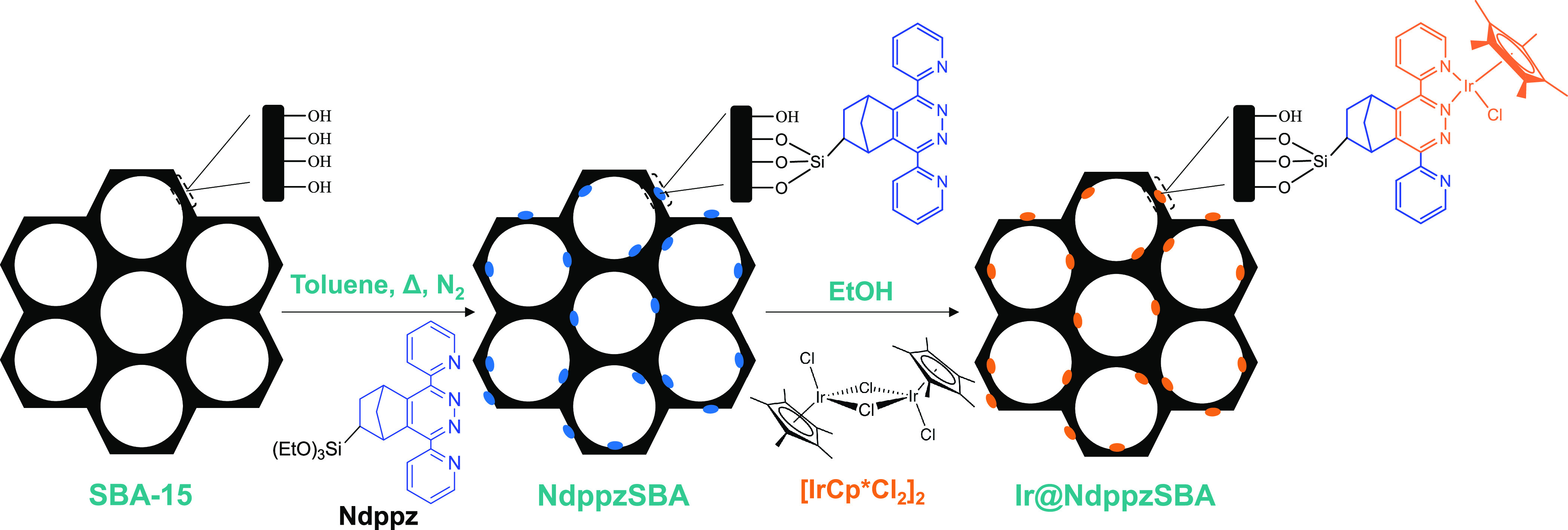
Synthetic Procedure for Ir@NdppzSBA

A two-step route has been carried out for the heterogenization of an iridium water oxidation catalyst (WOC) on a silica-based organic–inorganic hybrid material (Scheme 1). For that, a trialkoxysilane precursor, Ndppz, was synthesized following a click chemistry approach by an efficient inverse electron demand Diels–Alder reaction (iEDDA). Postsynthesis modification of a siliceous matrix such as SBA-15 by grafting with Ndppz afforded an organosilica with large pores, which integrated pendant dipyridyl-pyridazine adducts as N-chelating ligands with a great potential for coordination of several transition metals. Finally, metalation of the dipyridyl-pyridazine adducts with [IrCp*Cl_2_]_2_ resulted in a heterogeneous iridium bipyridine-like water oxidation catalyst for efficient oxygen evolution using CAN as an oxidizing agent in acidic conditions.

### 2.1 Characterization of the catalyst

**Figure 1:**
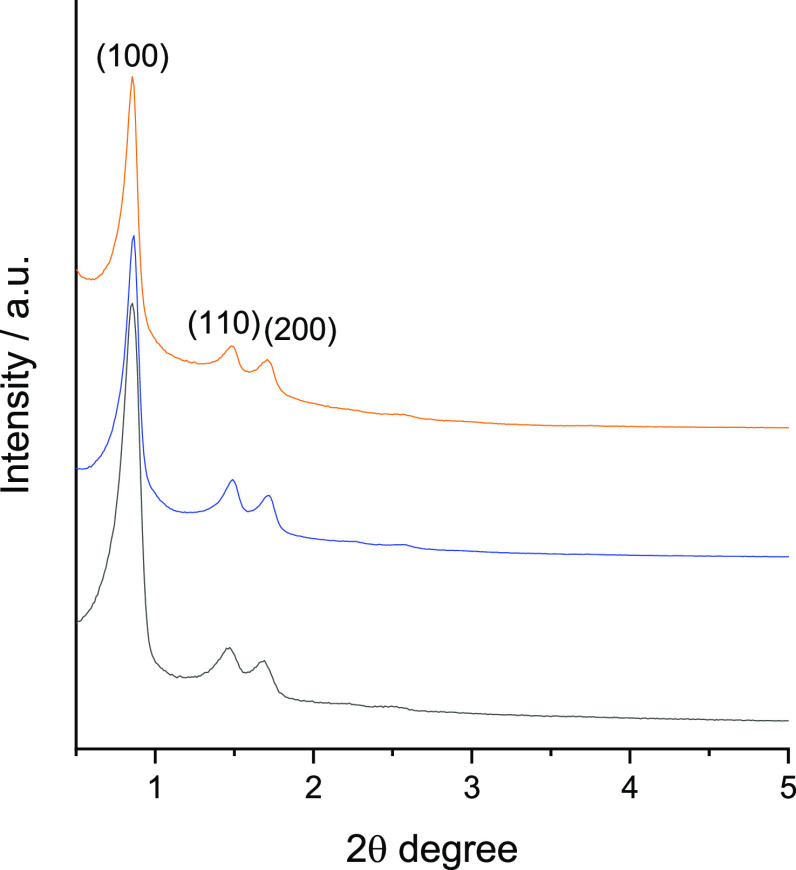
X-ray diffraction patterns of SBA-15 (black line), NdppzSBA (blue line), and Ir@NdppzSBA (orange line).

Low-angle X-ray diffraction patterns of SBA-15, NdppzSBA, and Ir@NdppzSBA are depicted in Figure 1. All diffractograms displayed one strong peak and two additional peaks of lower intensity at higher incidence angles related to typical lattice planes of a *P*6*mm* hexagonal arrangement structure,^38,39^ suggesting the preservation of the initial ordered mesostructure after postfunctionalization reactions. Although XRD patterns displayed analogous lattice planes, a decrease in the intensity of the signals was observed after the incorporation of Ndppz and the subsequent coordination of the iridium complexes, indicating differences in the scattering contrasts within the pores after the functionalization.^40^ The interplanar spacing (*d*_100_) of the parent material was calculated from Bragg’s Law, obtaining values of 10.3, 6.0, and 5.2 nm for reflections (100), (110), and (200), respectively, whereas its lattice parameter (*a*_0_) was estimated as 12.0 nm. Similar values of *d*_100_, and consequently of*a*_0_, were obtained for NdppzSBA and Ir@NdppzSBA. Conventional TEM images of SBA-15, NdppzSBA, and Ir@NdppzSBA revealed a hexagonal arrangement of uniform pores, indicating no influence of the functionalization processes on the highly ordered hexagonal mesostructured morphology (Figure S2). These results corroborated those obtained by XRD.

**Figure 2:**
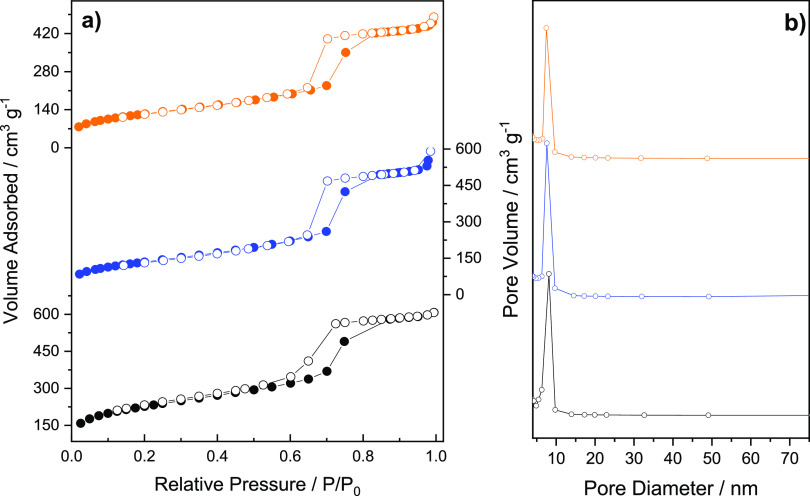
N_2_ adsorption–desorption isotherms (a) and pore size distributions (b) of SBA-15 (black symbols), NdppzSBA (blue symbols), and Ir@NdppzSBA (orange symbols).

Figure 2 shows nitrogen adsorption–desorption isotherms and pore size distributions for all of the synthesized materials. They exhibited type IV isotherms with an H1-type hysteresis loop and a sharp increase in adsorbed volume in the *P*/*P*_0_ range from 0.6 to 0.8, typical of mesoporous materials with large pores.^41^ The Brunauer–Emmett–Teller surface area (*S*_BET_), pore volume (*V*_P_) and pore diameter (*D*_P_) for the parent material were estimated as 817 m^2^ g^–1^, 1.02 cm^3^ g^–1^, and 8.1 nm, respectively. Condensation of Ndppz with the free silanols of SBA-15 led to a significant decrease in the textural properties, which dropped to 469 m^2^ g^–1^, 0.82 cm^3^ g^–1^, and 7.6 nm. Subsequent attachment of the Ir complex to the surface adducts led to an additional decrease in *S*_BET_ (433 m^2^ g^–1^), *V*_P_ (0.69 cm^3^ g^–1^), and *D*_P_ (7.5 nm). The reduction in the textural properties of the materials was accompanied by an increase in the wall thickness from 3.9 to 4.3 and 4.4 nm after the functionalization steps. These physical changes confirmed the successful postsynthesis modification of the parent material, which is in agreement with the trend revealed in different functionalization processes of periodic mesoporous organosilicas (PMOs) and metal organic frameworks (MOFs) reported by our group.^42−46^ All physicochemical properties of SBA-based synthesized materials are summarized in Table 1.

**Table 1:** Physicochemical Properties of SBA-Based Materials

**solid**	***S***_**BET**_**(m**^**2**^**g**^**–1**^**)**	***V***_**p**_^a^**(cm**^**3**^**g**^**–1**^**)**	***D***_**p**_^b^**(nm)**	***a***_**0**_^c^**(nm)**	**wall thickness**^d^**(nm)**
SBA-15	817	1.02	8.1	12.0	3.9
NdppzSBA	469	0.82	7.6	11.9	4.3
Ir@NdppzSBA	433	0.69	7.5	11.9	4.4

a BET surface areas were obtained from the adsorption branch.

b Pore volume and pore size distribution were calculated by analysis of the adsorption branch of the isotherms using the BJH method.

c Lattice parameter calculated by *a*_0_=2*d*_100_/.

d Wall-thickness estimated from *a*_0_**–***D*_p._

**Figure 3:**
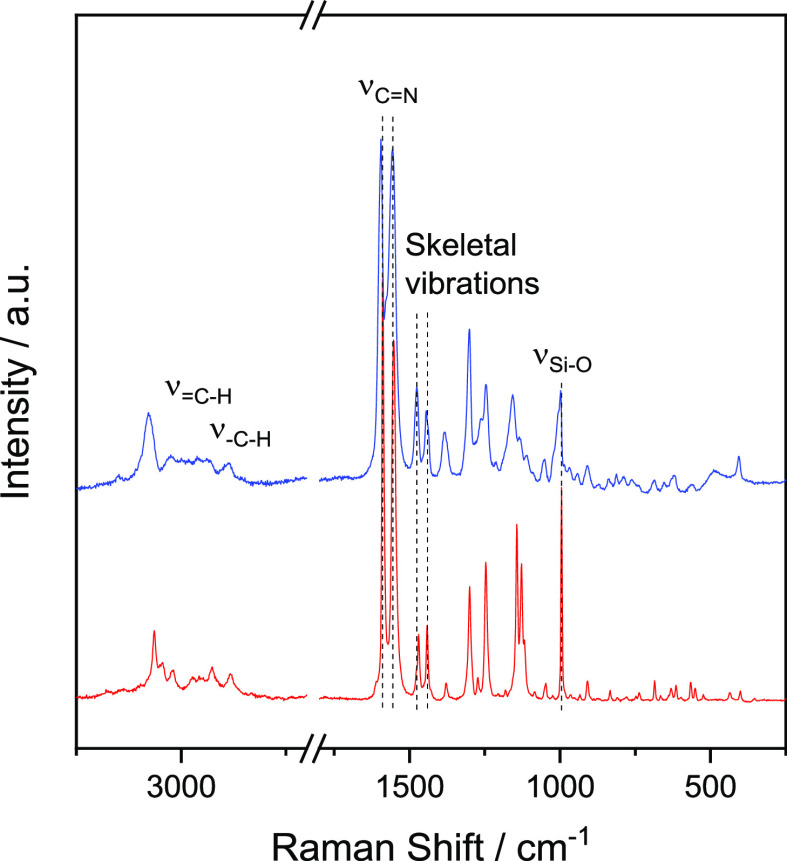
Raman spectra of Ndppz trialkoxysilane (red line) and NdppzSBA (blue line).

The covalent grafting of Ndppz in SBA-15 was also confirmed by Raman spectroscopy (Figure 3). The Raman spectrum of Ndppz trialkoxysilane showed a peak at 995 cm^–1^ assignable to the Si–O stretching vibration. Signals at 1555, 1444, and 1476 cm^–1^ were ascribed to skeletal vibrations of the pyridine/pyridazine heterocyclic rings.^43^ The intense band at 1590 cm^–1^ was attributed to C=N stretching modes.^47^ Additionally, several signals appeared in the region of 2800–3100 cm^–1^. Those signals located at Raman shifts higher than 3000 cm^–1^ were ascribed to the =C–H stretching of aromatic carbons from the dipyridyl-pyridazine moieties, whereas signals below 3000 cm^–1^ were assigned to C–H stretching of aliphatic carbons from the bicyclic structure. As can be seen, all signals present in the spectrum of the Ndppz precursor were also found in the Raman spectrum of NdppzSBA, thus corroborating the successful immobilization of Ndppz on the SBA surface.

**Figure 4:**
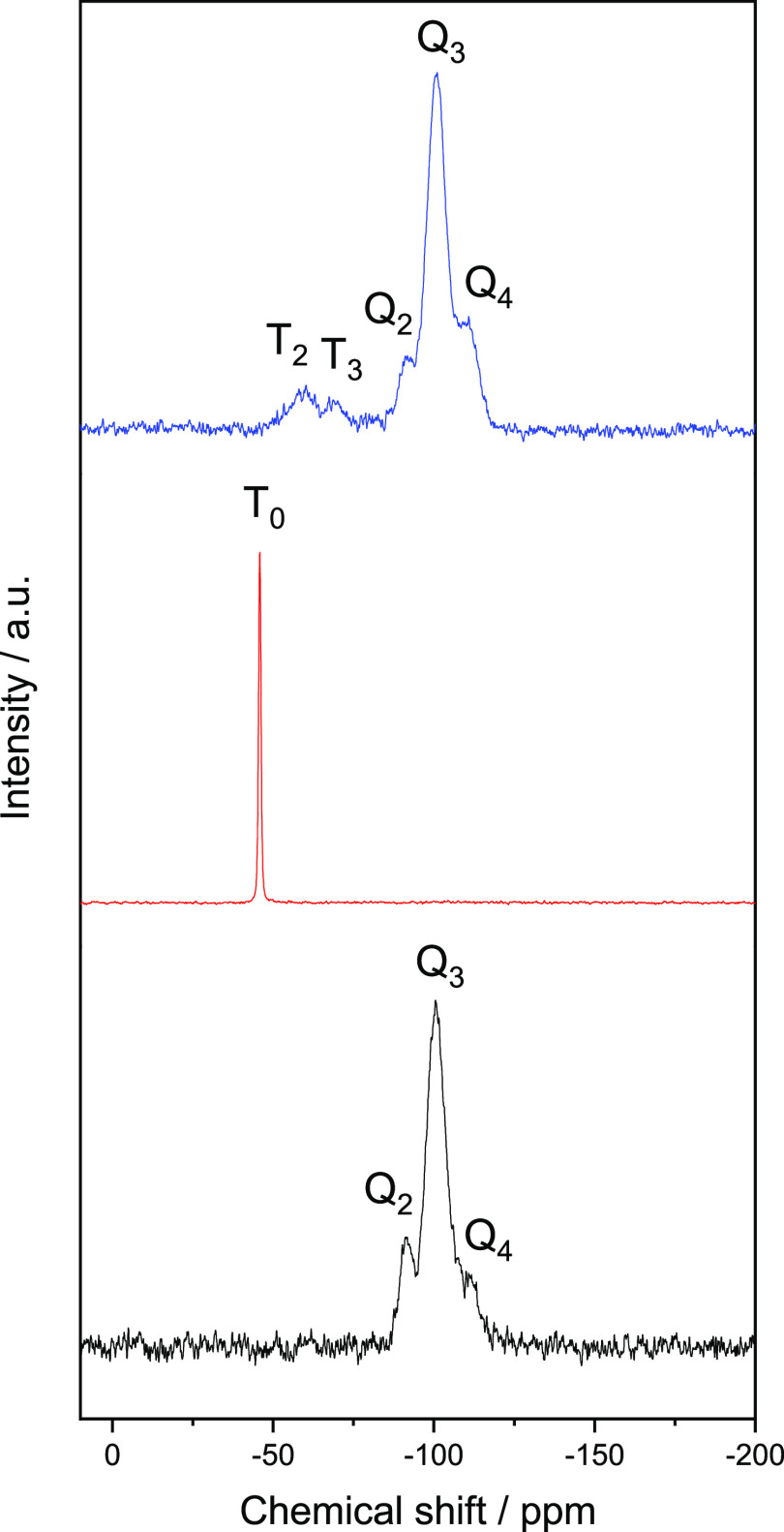
^29^Si CP/MAS NMR spectra of SBA-15 (black line), Ndppz (red line), and NdppzSBA (blue line).

Additionally, the Si environment was evaluated upon functionalization by solid-state ^29^Si NMR. The ^29^Si NMR spectra of the Ndppz organosilane precursor, SBA-15, and NdppzSBA samples exhibited different T_*n*_ and/or Q_*n*_ sites depending on the silane structure and the connectivity of the Si–O tetrahedra (Figure 4). SBA-15 showed the characteristic signals at −92, −101, and −110 ppm ascribed to Q_2_ [(SiO)_2_***Si***(OEt/OH)_2_], Q_3_ [(SiO)_3_***Si***(OEt/OH)], and Q_4_ [(SiO)_4_***Si***] units, respectively.^48^ In the case of Ndppz trialkoxysilane, a single narrow signal at −46 ppm was revealed, attributable to T_0_ sites [***Si***C(OEt/OH)_3_].^49^ Both T_*n*_ and Q_*n*_ sites were present in the NdppzSBA material. Whereas Q_2_, Q_3_, and Q_4_ signals appeared at the same chemical shift as for SBA-15, new signals at −59 and −69 ppm assigned to T_2_ [(SiO)_2_***Si***C(OEt/OH)] and T_3_ [(SiO)_3_***Si***C] sites,^48^ respectively, were obtained, confirming the effectiveness of the grafting process of the Ndppz organosilane precursor on the surface of the SBA-15 mesoporous silica.

**Figure 5:**
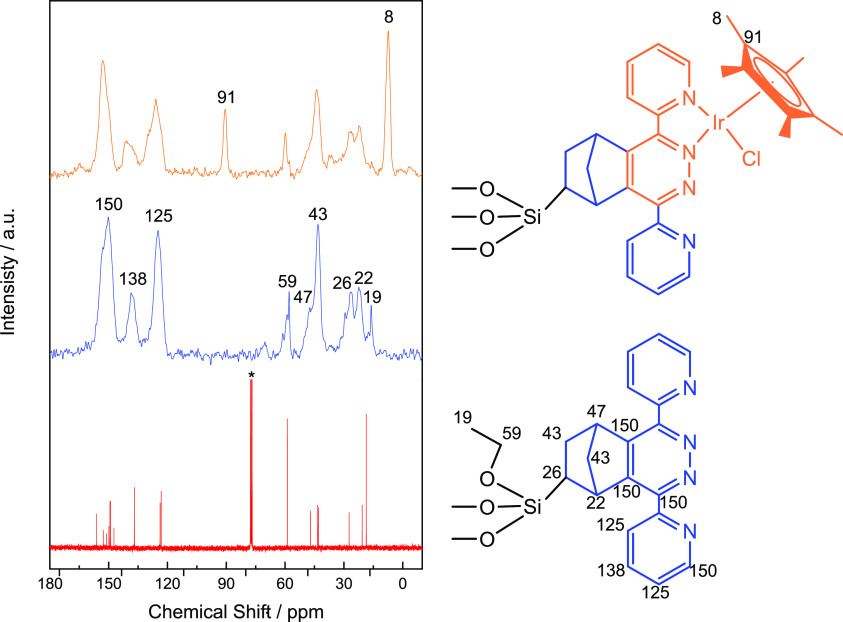
^13^C CP/MAS NMR spectra of Ndppz trialkoxysilane (red line), NdppzSBA (blue line), and Ir@NdppzSBA (orange line). The asterisk (*) denotes the CDCl_3_ solvent residual signal.

The coordination of the IrCp*Cl moieties to the N-chelating coordination sites provided by NdppzSBA was confirmed by different techniques, such as ^13^C CP/MAS NMR, UV/vis diffuse reflectance spectrometry, and X-ray photoelectron spectroscopy. Figure 5 shows ^13^C CP/MAS NMR measurements of the functionalized materials. The NdppzSBA spectrum revealed five signals between 20 and 50 ppm attributed to Csp^3^ of the norbornene ring.^45^ Downfield signals located at chemical shifts in the range of 120–160 ppm were characteristic of aromatic carbons from the dipyridyl-pyridazine adducts.^45^ Nonhydrolyzed ethoxy (−OCH_2_CH_3_) groups of the Ndppz precursor were evidenced by resonances at 19 and 59 ppm. After the attachment of the iridium complex, two new signals appeared at 8 and 91 ppm, corresponding to Csp^3^ (−Cp(CH_3_)_5_) and Csp^2^ (−Cp(CH_3_)_5_), respectively, of the Cp* ligand incorporated in the metalation process.^36^

**Figure 6:**
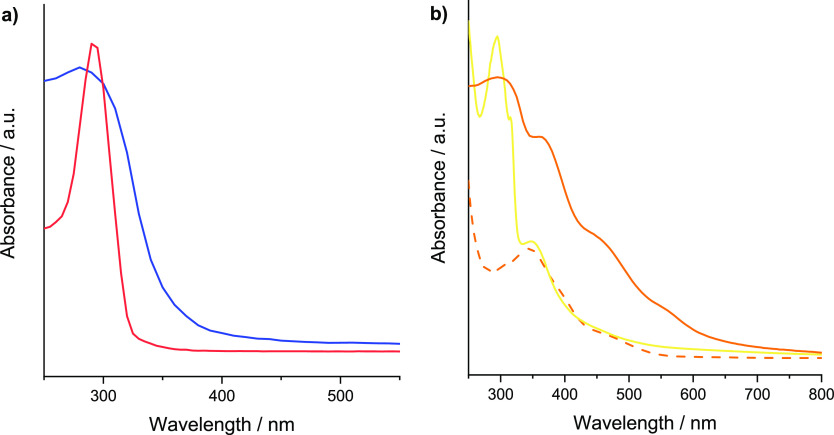
(a) UV–vis absorption spectrum of Ndppz (red line) in dichloromethane and UV–vis reflectance diffuse spectrum of NdppzSBA (blue line). (b) UV–vis absorption spectrum of [IrCp*Cl_2_]_2_ (orange dashed line) and [IrCp*Cl(bpy)]Cl (yellow line) in ethanol and UV–vis reflectance diffuse spectrum of Ir@NdppzSBA (orange solid line).

Covalent immobilization of the trialkoxysilane in SBA-15 was also corroborated by the presence of the π–π* transition at 280 nm,^50,51^ characteristic of dppz adducts present in the UV–vis reflectance diffuse spectrum of NdppzSBA (Figure 6a). The presence of [IrCp*Cl(dppz)] complexes anchored on NdppzSBA was further confirmed by the presence of new bands (Figure 6b). The absorption band in the UV region appeared shifted to 300 nm, suggesting interaction of the dppz coordination sites with the iridium center. Two additional bands at 360 and 450 nm were displayed after metalation attributed to metal-to-ligand charge transfer transitions (MLCTs).^52^ Moreover, a weak and long tail absorption at around 550 nm could be assigned to the direct spin-forbidden absorption from the singlet ground state to the triplet excited states similar to other Ir(III) bipyridine-based complexes.^53^

**Figure 7:**
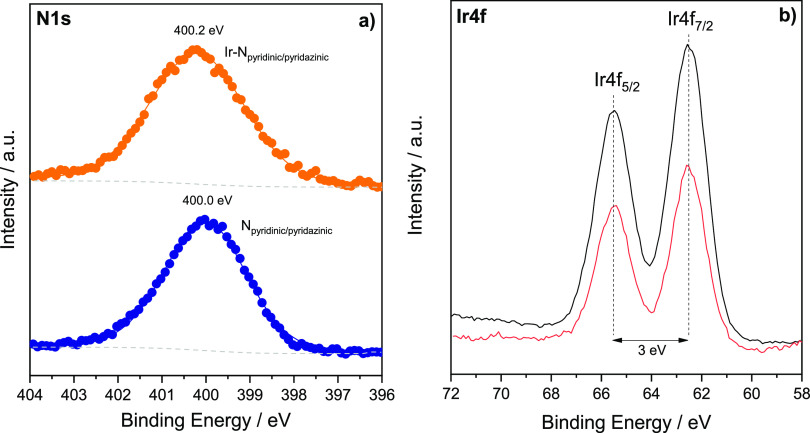
(a) N1s XPS spectra of NdppzSBA (blue symbols) and Ir@NdppzSBA (orange symbols). (b) Ir4f XPS spectra of [IrCp*Cl(bpy)]Cl (black line) and Ir@NdppzSBA (red line).

XPS surface analysis was carried out to confirm the complexation of iridium in the Ir@NdppzSBA sample and to analyze its oxidation state. Survey spectra of SBA-15 indicated the presence of Si and O in the sample (Figure S3). The Si2p region exhibited only a peak centered at 103.4 eV, whereas the O1s region showed two signals at 530.9 and 532.8 eV associated to Si–OH and Si–O–Si bonds, respectively.^54^ Concerning NdppzSBA, the survey spectrum of the sample showed peaks corresponding to C and N in addition to Si and O, which confirmed the incorporation of dipyridyl-pyridazine adducts (Figure S4). The C1s spectrum showed a lower binding energy peak at 285.3 related to C–H, C–C, and C_Ar_ and a higher binding energy peak at 286.7 eV associated with C=N, both characteristic of carbon species from Ndppz trialkoxysilane. An additional contribution at 288.8 eV was associated to the π–π* shakeup satellite peak,^55,56^ characteristic of delocalized π conjugation in pyridinic and pyridazinic aromatic rings of Ndppz. The N1s region showed only a contribution at 400.0 eV, suggesting no differences in binding energy between the pyridinic and pyridazinic nitrogens of the dppz adduct^57^ (Figure 7a). Surface analysis of Ir@NdppzSBA revealed the presence of Si, O, C, and N species apart from new Ir and Cl components related to the metalation of the iridium complex (Figure S5). The iridium metalation step was accompanied by a slight upshift in the original binding energy of the N1s spectrum (Figure 7a) to 400.2 eV, suggesting that the chemical environment had changed because of electronic interactions between iridium and the Ndppz chelating unit.^58^ Additionally, two peaks were observed at about 62.5 eV (Ir 4f 7/2) and 65.5 eV (Ir 4f 5/2) for Ir4f (Figure 7b), in good accordance to those of the homogeneous analogue, thus confirming the presence of trivalent iridium in the sample.^59−61^ The Cl2p spectrum was fitted into two peaks at 198.1 eV (Cl 2p 3/2) and 199.7 eV (Cl 2p 1/2), which corresponded to the chloride ligand incorporated in the metalation step (Figure S5e).

The nitrogen content in NdppzSBA was estimated by CHN elemental analysis as 0.462 mmol g^–1^, which means that 0.116 mmol g^–1^ of dppz was grafted on the silica support. Analysis of anchored iridium species on Ir@NdppzSBA by inductively coupled plasma-mass spectrometry revealed a loading of 0.044 mmol of Ir g^–1^, resulting in an Ir/dppz ratio of 0.38. Accordingly, a significant fraction of the dppz adducts remained uncoordinated, and the catalyst surface was decorated with isolated single-site mononuclear complexes, as previously reported by our group when 2,2’-bipyridine (bpy) and 2-phenylpyridine (ppy) ligands were present.^45^

### 2.2 Water oxidation reactions

**Figure 8:**
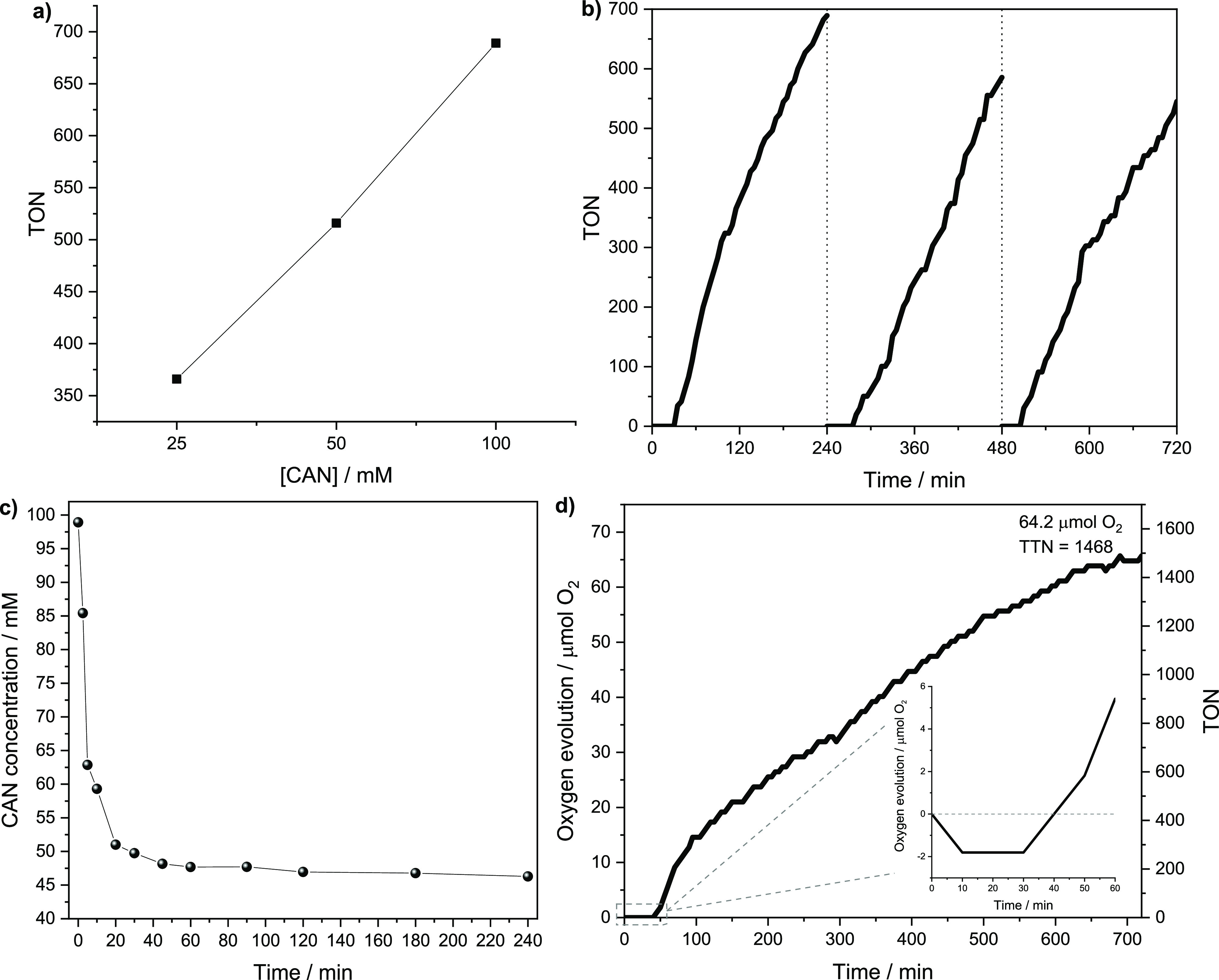
(a) Optimization of the CAN concentration for the oxygen evolution reaction. Experimental conditions: 25, 50, and 100 mM CAN in 10 mL of 0.1 M HNO_3_ solution using Ir@NdppzSBA as catalyst (1 mg, 0.044 μmol Ir) at 4 h reaction time; (b) time-dependent oxygen evolution curves for the Ir@NdppzSBA heterogeneous catalyst of three reuse reaction cycles of WOR under 100 mM CAN in 0.1 M HNO_3_ at room temperature; (c) time course of CAN consumption determined by UV–vis spectroscopy; and (d) long reaction time test for Ir@NdppzSBA, showing reaction profile at short times (inset).

Ir@NdppzSBA was evaluated as a heterogeneous catalyst for water oxidation reactions using Ce^4+^ (CAN) as the oxidant in an aqueous acid solution containing nitric acid (0.10 M, pH 1.0) at room temperature. O_2_ evolution was monitored by a gas pressure sensor every 5 min for 4 h. Gas phase analysis of the reaction flask headspace by GC chromatography revealed that oxygen was the only reaction product (Figure S6). First, the optimization of CAN concentration (Figure 8a) showed that oxygen generation increased linearly with oxidant concentration. This demonstrated that catalytic performance was not limited at high CAN concentrations where the catalyst was capable of catalytically producing more IrCp* active species during the catalytic water oxidation process.^62^ Subsequently, time-dependent oxygen evolution kinetics was carried out at a CAN concentration of 100 mM for three consecutive 4 h catalytic reaction cycles (Figure 8b). A remarkable stabilization period was observed until a positive variation in the gas phase pressure was detected in the headspace of the reactor. This fact could be explained by the formation of O_2_ bubbles after injection of the CAN solution.^25,63,64^ Thus, at a short time, a negative pressure is registered in our system, which is compensated over time when the evolved O_2_, first generated in the solution, is able to diffuse through the gas phase (Figure 8d inset). Although a plateau was not reached in the kinetic profile after 4 h of reaction, the catalyst was recovered to evaluate its reusability under the same experimental reaction conditions, showing a similar trend in the time course oxygen evolution in each cycle (Figure 8b). The heterogeneous system produced 30.1 μmol of O_2_ in the first run, which corresponded with a TON of 689 vs [Ir], whereas the initial TOF was calculated as 4.1 min^–1^. Under the same conditions, the absence of WOC resulted in zero activity due to the large activation barrier required to oxidize water, thus suggesting the requirement of a WOC to reduce this kinetic barrier.^65^ The catalytic activity of the heterogeneous system largely exceeded that obtained by the [IrCp*Cl(bpy)]Cl complex (119 turnovers) (Figure S7), comparatively showing an increase in catalytic activity per active site and, therefore, an efficient heterogeneous water oxidation catalysis. To further confirm the heterogeneous nature of the grafted [IrCp*Cl(dppz)] complex in the WOR, a leaching test was carried out (Figure S8). After the catalyst Ir@NdppzSBA was filtered, the reaction was left for another 4 h without observing additional oxygen production. The result of the leaching test was in agreement with the ICP-MS analysis of the reaction supernatant, which showed that a negligible amount of Ir species leached to the solution. Furthermore, the recovered catalyst after 4 h reaction was tested in a second and third reaction cycle. As can be seen in Figure 8b, the performance of the catalytic system slightly decreased with successive reaction cycles, showing TOF values of 3.4 min^–1^ for both cycles, thus confirming the remarkable stability and reusability of this heterogeneous catalyst.

The kinetics for the water oxidation reaction was further investigated by monitoring the CAN consumption by UV–vis spectroscopy at 275 nm (Figure S9, Figure 8). Figure 8c shows fast CAN consumption kinetics in the first hour, reaching a stabilized CAN depletion of 53% after 4 h of reaction. This behavior is in agreement with previous studies in which CAN depletion occurred faster than O_2_ evolution and showed an important consumption before detecting oxygen in the gas phase.^62^

In this water oxidation system, four molecules of CAN are consumed for the formation of a single oxidized aqueous O_2_ molecule. Accordingly, the oxygen evolution yield could be calculated by monitoring CAN depletion. Figure 8c shows 53% CAN depletion after 4 h reaction, whereas, at this time, 30.1 μmol of O_2_ was evolved, which corresponded to an oxygen yield of 23%. Additionally, the lifetime of the water oxidation system was evaluated at long reaction times (Figure 8d). After 12 h of reaction, a plateau was reached at 64.2 μmol of oxygen, which corresponded to a TTN of 1468. At this point, a 49% oxygen evolution yield was achieved. This moderate yield is probably due to the large excess of the oxidizing agent relative to the iridium active centers in the reaction. For comparison purposes, the catalytic activities of the Ir@NdppzSBA and other previously reported molecular [IrCp*Cl(bpy)]^+^ catalysts heterogenized in porous systems such as MOFs or silica-based organic–inorganic hybrid materials have been gathered in Table S1. Although reaction conditions are not identical, Ir@NdppzSBA is shown to be competitive in terms of TOF in relation with these heterogeneous catalysts.

The catalyst Ir@NdppzSBA was characterized after three WOR cycles. The UV–vis diffuse reflectance spectrum of Ir@NdppzSBA revealed the apparent loss of the metal-to-ligand charge transfer transitions at 360 and 450 nm (Figure S10). This significant change could be ascribed to the oxidative transformation of the Cp* ligands into acetic or formic acids, as previously described in homogeneous and heterogeneous WOCs.^30,34,36^ TEM images evidenced that the highly ordered hexagonal mesostructure remained even at high concentrations of CAN (Figure S11) and the formation of cerium oxide nanoparticles (ca. 20–25 nm) after WOR, as also reported by Inagaki et al.^36,66^ These nanoparticles showed a larger size than the pore size of the catalyst mesochannels (Figure S11b), producing pore blockage, which can explain the loss of activity after the first reaction cycle. The presence of ceria was also confirmed by Raman spectroscopy (Figure S12). The Raman spectrum displayed three main signals at 457, 605, and 1097 cm^–1^ attributed to symmetric Ce–O stretching vibration (F_2g_), defect-induced oxygen vacancies (D), and second-order longitudinal optical overtone (2LO) mode, respectively.^66,67^ The diffraction pattern at a low angle for the catalyst was preserved (Figure S13a). Additionally, the diffraction patterns of cerium oxide nanoparticles appeared at higher angles, mainly associated with the characteristic reflections of the cerianite crystalline phase (CeO_2_), thus confirming the deposition of the nanoparticles on the catalyst (Figure S13b).^68,69^ Surface analysis of the catalyst by XPS after reaction revealed Ce components, in addition to those elements previously mentioned for the Ir@NdppzSBA catalyst (Figure S14). Deconvolution of the Irf4 region indicated the preservation of the trivalent oxidation state of the iridium species (Figure S14b). Interestingly, the recovered catalyst showed a greenish color, which again became yellow after washing with water. These changes suggested the presence of iridium (IV) after the reaction, whereas the neutralization with deionized water showed that these species were unstable, returning to iridium (III), as previously reported.^34^ In addition, no particles of IrO_2_ were generated during the reaction due to the lack of Ir4f contributions at 61.6 ± 0.5 eV,^70^ as can be expected by the instability of IrO_2_ particles at pH = 1,^71^ thus corroborating the molecular catalytic nature of the WOR. The N1s spectrum (Figure S14c) revealed the presence of surface ammonium (402.0 eV) and nitrate species (407.1 eV) in the sample.^72,73^ Moreover, Cl2p components were not found, consistent with the WOR mechanism described in the literature (Figure S14d).^17,25−28^ The study of the Ce3d core level provided further information on the oxidation state of the Ce components, showing the coexistence of Ce(IV) and Ce(III) species on the surface of the catalyst (Figure S14e). The Ce3d XPS spectrum was fitted into eight contributions associated to four spin–orbit doublets. The presence of Ce(IV) was demonstrated based on three doublets identified as ν–u (3d_5/2_–3d_3/2_) at 883.0 and 901.3 eV, ν″–u″ at 888.3 and 907.3 eV, and ν‴–u‴ at 898.7 and 917.2 eV, whereas Ce(III) was ratified by a doublet, ν′–u′, at 885.8 and 904.1 eV.^74,75^

## 3 Conclusions

A new approach for obtaining a heterogeneous water oxidation catalyst through the immobilization of a IrCp* complex on N-chelating sites covalently incorporated into the surface of an SBA-15 mesoporous silica has been reported. The formation of [IrCp*Cl(dppz)]^+^ complexes on the pendant Ndppz units of the functionalized SBA-15 was confirmed through different techniques. The resulting heterogeneous catalyst Ir@NdppzSBA, with suitable iridium single sites for water oxidation reactions, was evaluated in strong acidic aqueous media using CAN as a sacrificial oxidizing agent. This catalyst showed a high stability for at least three reaction cycles. Thus, the catalyst gave a high total turnover number of 1468 until deactivation of the system. A comparative study with its homogeneous counterpart resulted in higher values of turnover numbers for the heterogeneous catalyst, indicating an enhanced conversion per active site in our heterogeneous WOC. The characterization of Ir@NdppzSBA after three reaction cycles proved the surface deposition of cerium oxide nanoparticles associated with the slight decrease in the catalytic performance in subsequent reaction cycles due to pore blockage during the reaction but without affecting the preservation of the hexagonal mesoporous structure of the WOC. These results evidenced that Ir@NdppzSBA was an efficient heterogeneous cerium(IV)-driven water oxidation catalyst and the suitability of NdppzSBA as a promising platform for heterogeneous metal-bipyridine-based catalytic systems and for the design of artificial photosynthesis devices.

## 4 Experimental section

### 4.1 Chemical and materials

Reagents and solvents were purchased from commercial sources and used without further purification. Hydrazine hydrate (50–60%), 2-pyridinecarbonitrile (99%), and sodium nitrite (97%) were purchased from Aldrich for the synthesis of 3,6-di-2-pyridyl-1,2,4,5-tetrazine (dptz). The organosilane precursor (Ndppz) was synthesized using 5-norbornen-2-yltriethoxysilane (97%, Fluorochem), 2,3-dichloro-5,6-dicyano-*p*-benzoquinone (DDQ, 98%, Aldrich), and anhydrous tetrahydrofuran (Aldrich). Pluronic P123 (EO_20_PO_70_EO_20_, average MW = 5800 g/mol, Aldrich), tetraethyl orthosilicate (98%, Acros Organics), and hydrochloric acid (37%, Labkem) were employed for the synthesis of the SBA-15 material. Toluene (anhydrous, 99.8%, Aldrich) was used as the solvent for Ndppz grafting over SBA-15. postsynthetic Ir metalations were carried out using pentamethylcyclopentadienyliridium(III) dichloride dimer (97%, TCI), dry ethanol (Supelco), and *N*,*N*-dimethylformamide (Pure, Panreac). Ammonium cerium(IV) nitrate (CAN, 98.5%, Aldrich) and nitric acid (69%, Panreac) were used in oxygen evolution reactions.

### 4.2 Characterization techniques

X-ray diffraction patterns (XRD) were recorded on a Bruker D8 Discover A25 diffractometer at 40 kV and 30 mA (Cu Kα radiation, λ = 0.154 nm). Diffractograms were collected in the range of 0.5 < 2θ < 5. Nitrogen adsorption and desorption experiments were performed at 77 K using a Micromeritics ASAP 2010 instrument. Samples were previously outgassed at 120 °C overnight prior to analysis. The surface area was determined using the BET method, and pore size distribution was obtained by analysis of the adsorption branch of the isotherms using the Barrett–Joyner–Halenda (BJH) method. Structural analyses were accomplished by TEM using a JEOL JEM 1400 microscope operating at 300 kV. The preparation of the samples was carried out by direct deposition of the powders on the carbon-coated copper grids (Agar Scientific Ltd.). Raman spectroscopy was performed with an argon laser (λ = 532 nm) on a Renishaw Raman spectrometer (InVia Raman Microscope) equipped with a Leica microscope and a Renishaw CCD Camera (578 × 400). The solid-state ^13^C and ^29^Si CP/MAS NMR measurements were performed on a Bruker Avance III HD 400 WB spectrometer operating at 100.61 and 79.49 MHz, respectively. Tetramethylsilane (TMS) standard was employed as the chemical shift reference. UV–vis and UV–vis diffuse reflectance measurements were taken using a Perkin-Elmer Lambda 650 S UV/vis spectrometer, which operates in a double beam mode with a 150 mm integrating sphere in a wavelength range between 250 and 700 nm. A quartz cuvette was used, and the recorded absorbance values were corrected for background noise and solvent absorbance. XPS measurements were acquired with a SPECS PHOIBOS150 MCD X-ray photoelectron spectrometer. Pellet-shaped samples were analyzed after an outgassing step in ultrahigh vacuum. The X-ray source was a monochromatic Al anode (1486.7 eV). Accurate binding energies were determined with respect to the position of Si2p at 103.4 eV. The loading of grafted Ndppz groups was determined using elemental analysis. CHNS elemental analysis was performed on a LECO TRUSPEC CHNS MICRO elemental analyzer, where samples were combusted three times at a temperature below 1050 °C. Quantification of iridium was carried out by inductively coupled plasma mass spectrometry (ICP-MS) using a Perkin-Elmer NexION 350X spectrometer.

### 4.3 Synthesis of materials

#### Synthesis of NdppzSBA

SBA-15 was synthesized according to a procedure described in the literature.^76^ Ndppz was synthesized following a procedure previously reported by our group.^45^ The Ndppz organosilane precursor was grafted onto SBA-15 following the method of Lauwaert et al. with a slight modification.^78^ Before grafting, SBA-15 was heated at 150 °C under a vacuum for 24 h to remove any adsorbed water. Subsequently, a 100 mL three-neck round-bottom flask connected to a Schlenk system was charged with 500 mg of SBA-15. Then, the flask was filled with nitrogen, and a Ndppz solution (0.1 mmol, 46 mg) in anhydrous toluene (21 mL) was injected. The resulting mixture was stirred and heated to reflux overnight. The solid was collected by filtration, washed with chloroform to remove unreacted Ndppz organosilane, and dried under a vacuum at 100 °C. The resulting pale pink powder was denoted as **NdppzSBA**.

#### Synthesis of [IrCp*Cl(bpy)]Cl

The homogeneous [IrCp*Cl(bpy)]Cl complex was synthesized by scaling the Youinou et al. procedure.^77^ 2,2’-Bipyridine (122.5 mg, 0.78 mmol) was dissolved in DMF (2.5 mL) and added to a solution of [IrCp*Cl_2_]_2_ (250 mg, 0.3 mmol) in DMF (25 mL). The resulting mixture was stirred under nitrogen for 5 h. A change in the color from orange to yellow was observed during the reaction. Then, the volume of the solution was halved by vacuum distillation. Subsequent addition of diethyl ether resulted in the precipitation of the iridium complex as the chloride salt. The precipitate was washed with diethyl ether and hexane and recrystallized from acetonitrile/diethyl ether, yielding 129 mg (38%) of a yellow solid. The IrCp*Cl(bpy) complex was used in homogeneous catalytic water oxidation reactions without further purification. UV–vis λ_max_, nm: 295, 315, 347, and 421.

#### Synthesis of Ir@NdppzSBA

A 100 mL round-bottom flask was charged with NdppzSBA (150 mg), [IrCp*Cl_2_]_2_ (0.012 mmol, 10 mg), and 30 mL of dry ethanol.^36^ The mixture was stirred and heated at reflux overnight. The resulting solid was collected by filtration, washed with *N*,*N*-dimethylformamide and distilled water, and dried under a vacuum at 100 °C. A change in its color suggests the correct metalation with iridium complexes, yielding **Ir@NdppzSBA** as a pale orange powder.

### 4.4 Experimental conditions of water oxidation reactions

In a typical experiment, 1 mg of Ir@NdppzSBA and a magnetic stirrer were introduced in a two-neck round-bottom flask connected to a switchable three-way valve through a Torion screw. The system was purged and charged with an inert gas atmosphere through three vacuum/nitrogen cycles. In parallel, a solution of CAN containing nitric acid (0.10 M, 25 mL, pH = 1.0) was deoxygenated by bubbling N_2_ into the solution for 5 min. Then, 10 mL of the CAN solution was injected with a syringe into the reactor. The resulting mixture was stirred at 25 °C. After the reaction, the suspension was centrifuged to recover the catalyst, which was washed with deionized water. For further reaction cycles, the catalyst was dried under a vacuum and reused without further purification under identical reaction conditions.

Oxygen evolution reactions were recorded by triplicate through monitorization of gas phase pressure variations every 5 min inside the closed reactor vessel using a Man on the Moon series X103 gas evolution kit (Figure S1). The reaction flask is connected to a switchable three-way valve allowing the possibility of connecting the reactor vessel to the exterior to be used as a conventional Schlenk flask or connecting the flask to the pressure transducer so that the system is closed. The data processing was carried out assuming oxygen as an ideal gas.

Conversion to oxygen was expressed as follows: the catalytic activity of the Ir-WOC was expressed as turnover number (TON, mol of O_2_ produced per mol of Ir); the initial rate of the WOR was evaluated in terms of turnover frequency (TOF, mol of O_2_ produced per mol of Ir per unit time); and the degradation of the catalytic system was measured with the total turnover number (TTN, as mol of O_2_ produced per mol of Ir during the water oxidation system lifetime)

### 4.5 Recycling of catalyst

After the catalytic experiment, the reaction mixture was suspended in water (10 mL) inside a Falcon tube (15 mL) and centrifuged (10,000 rpm, 10 min). The supernatant was removed, and the catalyst was resuspended in water (10 mL) and centrifuged (10,000 rpm, 10 min). The resulting solid was dried under a vacuum overnight.

## Supporting Information Available

Illustration of the different components contained in the Man on the Moon series X103 gas evolution kit, supplementary characterization of the synthesized materials (SBA, NdppzSBA, and Ir@NdppzSBA), as well as the postreaction characterization of Ir@NdppzSBA, headspace reaction vessel chromatograms before and after water oxidation reaction, kinetic water oxidation curve for the homogeneous [IrCp*Cl(bpy)]Cl complex, leaching test of the Ir@NdppzSBA catalyst, and calibration curve of CAN obtained from UV–vis spectra (DOCX)

## Notes

^†^ Deceased May 12, 2023
