# Supplementary material for: Iridium-Complexed Dipyridyl-Pyridazine Organosilica as a Catalyst for Water Oxidation
Source: Inorg Chem. 2023 Jul 17;62(30):11954–65. doi: 10.1021/acs.inorgchem.3c01386 (PMC10394666; doi:10.1021/acs.inorgchem.3c01386)
Supplement: Supplementary file 1 — ic3c01386_si_001.docx [file ic3c01386_si_001.docx]

Supporting Information

**IRIDIUM-COMPLEXED DIPYRIDYL-PYRIDAZINE ORGANOSILICA AS CATALYST FOR WATER OXIDATION**

Raúl Rojas-Luna^a,‡^, Juan Amaro-Gahete^a,‡^, César Jiménez-Sanchidrián^a†^, José Rafael Ruiz^a^, Dolores Esquivel^a*^, Francisco José Romero-Salguero^a*^.

^a^Departamento de Química Orgánica, Instituto Químico para la Energía y el Medioambiente (IQUEMA), Facultad de Ciencias, Universidad de Córdoba, Campus de Rabanales, Edificio Marie Curie, E-14071 Córdoba, Spain.

^‡^These authors contributed equally to this work

^†^Deceased May 12, 2023

*Corresponding authors: [q12esmem@uco.es](mailto:q12esmem@uco.es) (D.E.), [qo2rosaf@uco.es](mailto:qo2rosaf@uco.es) (F.J.R-S)

 **Figure S1.** Man on the Moon series X103 gas evolution kit.

**Figure S2**. TEM image of: (a,b) SBA-15, (c,d) NdppzSBA and (e,f) Ir@NdppzSBA. Insets at higher magnification (f) evidence the preservation of the highly ordered hexagonal mesostructure. TEM images were acquired with the pore channel axis orientated parallel and perpendicular to the electron beam.

**Figure S3**. XPS spectra of SBA-15: (a) survey scan, (b) Si2p and (c) O1s.

**Figure S4**. XPS spectra of NdppzSBA: (a) survey scan, (b) Si2p, (c) O1s and d) C1s.

**Figure S5**. XPS spectra of Ir@NdppzSBA: (a) survey scan, (b) Si2p, (c) O1s, d) C1s and e) Cl2p.

**Figure S6.** GC-Chromatogram of the reaction flask headspace at initial time (pink line) and after 4 h reaction (dark line). Residual oxygen at initial time is ascribed to gas phase volume in the syringe needle.

**Figure S7.** Time dependence oxygen evolution curve for homogeneous [IrCp*Cl(bpy)]Cl complex under 100 mM CAN in 0.1 M of HNO_3_ at room temperature.

**Figure S8**. Leaching test for iridium species. The catalyst was filtered after 4 h of reaction, showing no additional oxygen evolution (red dashed line) compared to a typical WOR (black line). Reaction conditions: Catalyst (1 mg), 100 mM CAN in 10 mL HNO_3_ (pH=1).

**Figure S9**. a) UV-Vis spectra for CAN aqueous solutions at different concentrations from 0 to 100 mM, and b) Calibration graph obtained from UV-vis spectra of CAN aqueous solutions at the maximum absorption wavelength (λ_max_ = 275 nm).

**Figure S10**. UV-vis diffuse reflectance spectrum of Ir@NdppzSBA prior to WOR (red line) and after three reaction cycles (black line).

**Figure S11**. TEM images of Ir@NdppzSBA after three WOR cycles in a perpendicular (a) and parallel (b) orientation with respect to the electron beam.

**Figure S12**. Raman spectrum of Ir@NdppzSBA after three WOR cycles.

**Figure S13**. X-ray diffraction patterns for Ir@NdppzSBA after three WOR cycles at a) low-angle (0.5 - 5º) and (b) wide-angle (10 - 80º). XRD pattern of cerianite phase (red solid line) extracted from RRUFF database is also displayed.

**Figure S14**. XPS spectra for Ir@NdppzSBA after three reaction cycles: (a) survey scan, (b) Ir4f, (c) N1s, d) Cl2p and e) Ce3d.

**Table S1.** Comparison of the cerium-driven water oxidation performance of Ir@NdppzSBA with the heterogeneous [IrCp*Cl(bpy)]^+^ molecular catalysts reported in literature.

| **Catalyst** | **Support** | **Ir loading**  **(mmol Ir g^-1^)** | **TOF / min^-1^** | | | **Ref.** |
| --- | --- | --- | --- | --- | --- | --- |
|  |  |  | **3 mM CAN** | **15 mM CAN** | **100 mM CAN** |  |
| Ir-MOF | MOF | - | 0.12 | - | - | ^1^ |
| Ir-BPy-NT | Organosilica nanotubes | 0.32 | 0.77 | - | - | ^2^ |
| Ir_0.03_-BPy-PMO | PMO | 0.10 | 2.8 | - | - | ^3^ |
| Ir_0.07_-BPy-PMO | PMO | 0.22 | 2.5 | 4.6 | - |  |
| Ir_0.16_-BPy-PMO | PMO | 0.51 | 2.1 | - | - |  |
| Ir-bpy-MCM-41 | MCM-41 | 0.37 | 0.2 | - | - |  |
| Ir-BPy1/10-MS | Organosilica | 0.06 | 1.1 | - | - |  |
| Ir-BPy-NT | Organosilica nanotubes | 0.17 | 1.2 | 3.7 | - | ^4^ |
| Ir-BPy-SBA-15 | SBA-15 | - | 0.8 | 1.9 | - |  |
| Ir-Gbpy-NT | Organosilica nanotubes | - | 0.6 | 1.7 | - |  |
| Ir@NdppzSBA | SBA-15 | 0.044 | - | - | 4.1 | This work |

**References**

(1)	Wang, C.; Wang, J.-L.; Lin, W. Elucidating Molecular Iridium Water Oxidation Catalysts Using Metal–Organic Frameworks: A Comprehensive Structural, Catalytic, Spectroscopic, and Kinetic Study. *J. Am. Chem. Soc.* **2012**, *134* (48), 19895–19908. https://doi.org/10.1021/ja310074j.

(2)	Liu, X.; Goto, Y.; Maegawa, Y.; Ohsuna, T.; Inagaki, S. Mesoporous Organosilica Nanotubes Containing a Chelating Ligand in Their Walls. *APL Mater.* **2014**, *2* (11), 113308. https://doi.org/10.1063/1.4898195.

(3)	Liu, X.; Maegawa, Y.; Goto, Y.; Hara, K.; Inagaki, S. Heterogeneous Catalysis for Water Oxidation by an Iridium Complex Immobilized on Bipyridine‐Periodic Mesoporous Organosilica. *Angew. Chemie Int. Ed.* **2016**, *55* (28), 7943–7947. https://doi.org/10.1002/anie.201601453.

(4)	Zhang, S.; Wang, H.; Li, M.; Han, J.; Inagaki, S.; Liu, X. Enhanced Durability of an Iridium-Bipyridine Complex Embedded into Organosilica Nanotubes for Water Oxidation. *Dalt. Trans.* **2017**, *46* (29), 9369–9374. https://doi.org/10.1039/C7DT02026K.
